# What does the patient have to say? Valuing the patient experience to improve the patient journey

**DOI:** 10.1186/s12913-021-06341-3

**Published:** 2021-04-15

**Authors:** Raffaella Gualandi, Cristina Masella, Michela Piredda, Matteo Ercoli, Daniela Tartaglini

**Affiliations:** 1 Campus Bio-Medico di Roma University, Rome, Italy; 2 Politecnico di Milano, Milan, Italy; 3 Research Unit Nursing Science, Campus Bio-Medico di Roma University, Rome, Italy

**Keywords:** Patient-reported data, Patient experience, Patient journey, Process improvement

## Abstract

**Background:**

Patient-reported data—satisfaction, preferences, outcomes and experience—are increasingly studied to provide excellent patient-centred care. In particular, healthcare professionals need to understand whether and how patient experience data can more pertinently inform the design of service delivery from a patient-centred perspective when compared with other indicators. This study aims to explore whether timely patient-reported data could capture relevant issues to improve the hospital patient journey.

**Methods:**

Between January and February 2019, a longitudinal survey was conducted in the orthopaedics department of a 250-bed Italian university hospital with patients admitted for surgery; the aim was to analyse the patient journey from the first outpatient visit to discharge. The same patients completed a paper-and-pencil questionnaire, which was created to collect timely preference, experience and main outcomes data, and the hospital patient satisfaction questionnaire. The first was completed at the time of admission to the hospital and at the end of hospitalisation, and the second questionnaire was completed at the end of hospitalisation.

**Results:**

A total of 254 patients completed the three questionnaires. The results show the specific value of patient-reported data. Greater or less negative satisfaction may not reveal pathology-related needs, but patient experience data can detect important areas of improvement along the hospital journey. As clinical conditions and the context of care change rapidly within a single hospital stay for surgery, collecting data at two different moments of the patient journey enables researchers to capture areas of potential improvement in the patient journey that are linked to the context, clinical conditions and emotions experienced by the patient.

**Conclusion:**

By contributing to the literature on how patient-reported data could be collected and used in hospital quality improvement, this study opens the debate about the use of real-time focused data. Further studies should explore how to use patient-reported data effectively (including what the patient reports are working well) and how to improve hospital processes by profiling patients’ needs and defining the appropriate methodologies to capture the experiences of vulnerable patients. These topics may offer new frontiers of research to achieve a patient-centred healthcare system.

**Supplementary Information:**

The online version contains supplementary material available at 10.1186/s12913-021-06341-3.

## Background

Patient-reported data (satisfaction, preferences, outcomes and experience) have been increasingly studied with the aim of providing excellent patient-centred care [1, 2]. In particular, the collection of patient experience data is emerging as an increasingly key component in assessing the quality of delivered health services [3, 4]. Some authors have emphasised that understanding the patient experience represents an opportunity to design healthcare service delivery [5, 6]. However, healthcare professionals need to understand whether and how patient experience data can inform the design of service delivery from a patient-centred perspective more pertinently than other indicators [7–10].

Studies in the service management literature have shown that it is possible to understand the experience starting from the customer journey. The term ‘customer journey’ refers to ‘the processual and experiential aspects of service processes as seen from the customer viewpoint’ [11]. Kankainen et al. [12] describe it as ‘the process of experiencing service through different touchpoints from the customer’s point of view’. Customer experience is shaped before, during and after interactions with the service provider. Moving from services to healthcare, the experience of care is not only a matter of interaction but a multifaceted and complex phenomenon in which the health status, the context of care and presence of different health staff play an important role in achieving clinical outcomes [9, 13].

In the hospital context, the requirements of responding rapidly to the acute needs of patients through the integration of multiple actors and services increases this complexity. Timely movement of patients from one service to another is a necessary condition both for managing the volume of patients with different pathologies and for obtaining better clinical outcomes. Consequently, the patient experience of care and service delivery is the result of many successive touchpoints across services to receive care from different units, the totality of which constitutes the patient journey. Because on an individual level any experience is subjective, dynamic and context dependent [14], patient experience data collected at different points of the journey should make it possible to evaluate if there are discontinuities within the hospital units (e.g., inpatient ward) and between the different units (e.g., between hospital wards and operating rooms) crossed by the patient journey. Inter- and cross-organisational gaps such as obstructed data flow, unavailability of relevant information at points of intervention and a lack of services synchronisation may occur when a complete and consequential view of the whole process is missing. However, few studies have analysed how to improve the patient journey by starting from the patient experience of the service provided [15]. In particular, most of them focus only on a single step of the hospital journey without identifying which are the meaningful touchpoints for the patient [16–18]. Indeed, if on the one hand, the patient’s stay is itself composed of multiple steps within the hospital, the hospital journey is part of a larger patient journey, which extends further in time before and after hospitalisation. This is particularly the case for patients who have to undergo surgery, for which clinical examinations are required before admission and a follow-up is scheduled after discharge.

Furthermore, it is not yet clear what the best method is for collecting patient experience data throughout the patient journey [19]. A recent study analysed the hospital stay experience through the use of unstructured diaries completed in a patient’s own words. However, if, on the one hand, the authors confirm that it is possible to collect valuable data for the improvement of the service directly from the patient, then, on the other hand, the education level, age and clinical conditions could be a limit in understanding the experience of fragile patients [20].

The goal of the current study is twofold: 1) explore which data collected directly from the patient could be useful in improving the patient journey and 2) to analyse whether gathering timely patient experience data at different points of the patient journey within the hospital can capture areas for improvement in the patient journey.

## Methods

### Design and setting

A longitudinal survey was conducted in the orthopaedics department of a 250-bed Italian university hospital between January and February 2019. The unit of analysis was the journey of the orthopaedic patient from the first outpatient visit to hospital discharge. Accordingly, all patients who underwent major or minor orthopaedic surgery during the time period were considered for inclusion. The type of surgery and stage of the patient journey formed the analysis groups. The study was part of a larger hospital project to redesign the orthopaedic patient journey for hip or knee replacement surgery, here starting with the patient experience [19, 21]. In particular, the data collected by the hospital management to assess the quality of the service and that are presented in this work were integrated with interviews and the shadowing of patients, whose results are reported in other papers. The entire project received ethical approval from the organisation’s Ethics Committee (Protocol n.: 25/16 OSS ComEt CBM).

The orthopaedics unit has 34 beds for ordinary hospitalisation or day surgery and is divided into two multispecialty wards: one for ordinary admissions and one mainly for day surgery recovery. Some of the healthcare staff working within the various services are specialised, and a large part is composed of staff in training (residents and degree course students of medicine, nursing and physiotherapy). A centralised team that includes administrative staff and bed manager nurses handles the admissions calls and reception procedures.

### Reference terminology

To conduct the present research, the authors employed the following terms with corresponding meanings:

- Patient-reported data: views and opinions of patients on the care and on the service they have experienced.
- Patient satisfaction: ‘the extent to which the patient’s expectations were fulfilled’ [22].
- Patient experience: ‘the sum of all interactions, shaped by an organisation’s culture, that influences patient perceptions, across the continuum of care’ [23].
- Patient preference: ‘statements made by the patients regarding the relative desirability of a range of health experiences, treatment options and health states’ [24].
- Patient outcomes: ‘any report of the status of a patient’s health condition that comes directly from the patient, without interpretation of the patient’s response by a clinician or anyone else’ [25].

### Instruments

The current study was carried out with two different paper-and-pencil questionnaires delivered to the same patients at two different stages of the hospital patient journey. The first questionnaire was developed on purpose by the authors and completed by the patients at the time of admission to the hospital and at the end of hospitalisation. An English language version of the questionnaire is available as a supplementary file to this paper (see Additional file 1). The second questionnaire was the patient satisfaction questionnaire adopted by the hospital and completed by the patients at the end of hospitalisation. In this way, patient-reported outcomes (PRO), patient-reported experience (PRE), patient-reported satisfaction (PRS) and patient-reported preferences (PRP) were collected and analysed.

**Fig. 1:**
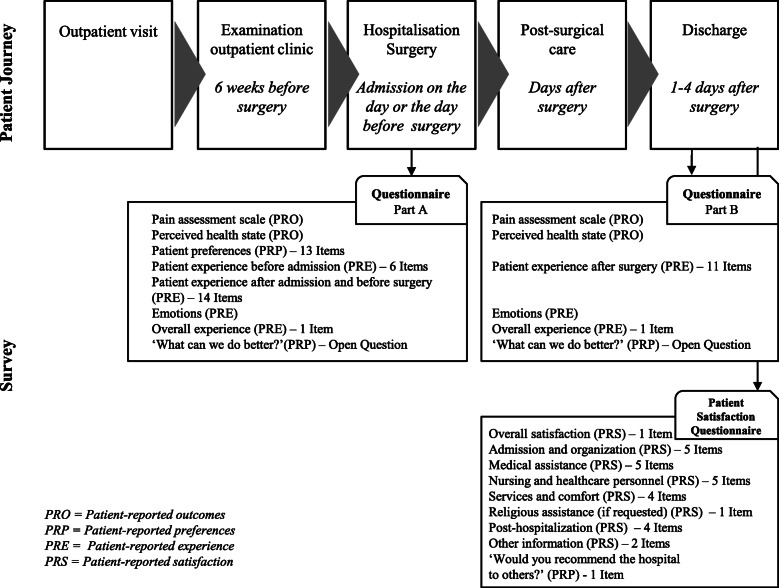
Patient journey and data collection

Figure 1 summarises the points of the patient journey where the data were collected and the focus of each questionnaire.

Consistent with the need to capture patient-reported data during a relatively rapid surgical pathway, the researchers chose to develop a questionnaire focused on the key themes that emerged from the results of the qualitative study previously conducted [19]. The questionnaire was developed by the first three authors to capture data on the following patient journey touchpoints: preadmission, admission to hospital, preparation for surgery, the postsurgery period and discharge. The purposes were the following: to create a questionnaire that is easy to read and fill in by the patient and to be administered at two key moments of the journey (at the time of admission before surgery and at discharge); to make the data more easily comparable between the different types of patient-reported data; to minimise the risk of patients not completing the questionnaire because of the high number of questions [26]; and to avoid less data being recorded in the case of elderly or low-educated patients [20].

The questionnaire items were identified to cover all the service quality dimensions indicated by Dagger [27] and Gustavsson [28]: interpersonal quality, technical quality, environmental quality, administrative quality, family quality and involvement quality. In addition, the international literature was consulted by the first and the second authors to develop a set of items evaluating the patient perspective on the level of importance of the different issues related to the hospital journey.

After a discussion between the authors, the questionnaire resulted in 37 closed items and one open question to be administered when the patient entered the hospital ward (Part A) and 15 closed items and one open question when the patient was discharged (Part B). The answers to the closed questions were possible within a 5- or 10-point Likert scale (depending on the items), on outcomes, preferences, experience and satisfaction.

Part A, which was administered upon arrival in the patient ward, included the following sections:

i) Pain assessment scale (0 = absent; 10 = the strongest pain) and perceived health state (0 = not satisfied at all; 5 = very satisfied);
ii) Patient preferences: evaluation of the self-perceived impact of the different issues related to the hospital journey on the patient’s life (e.g., instructions on how to get to the hospital or in case of waiting; not feeling pain; trusting professionals, etc.) – 13 items (0 = not at all important; 5 = very important);
iii) Patient experience before admission to hospital (e.g., visit, examinations in preparation for the surgery, etc.) – 6 items (scale of five values represented by emoticons  = bad experience;  = great experience)
iv) Patient experience after admission to hospital and before surgery (e.g., being involved in decisions about his/her care, being able to have family members nearby, etc.) – 14 items (scale of five values represented by emoticons  = bad experience;  = great experience);
v) Positive or negative emotions experienced at the moment of completing the questionnaire by choosing the main ones from the Plutchik’s wheel: serenity, trust, anticipation, apprehension, fear and anger.
vi) Overall experience up to the moment of filling in the questionnaire – one item (scale of five values represented by emoticons  = bad experience;  = great experience)
vii) A final open question: ‘What can we do better?’

Part B, administered upon discharge from the hospital ward, included the following sections:

i) Pain assessment scale (0 = absent; 10 = the strongest pain) and perceived health state (0 = not satisfied at all; 5 = very satisfied);
ii) Patient experience after surgery (e.g., usefulness of the instructions received on the clinical path; being involved in decisions about his/her care, being able to have family members nearby, etc.) – 11 items (scale of five values represented by emoticons  = bad experience;  = great experience);
iii) Positive or negative emotions experienced at the moment of completing the questionnaire by choosing the main ones from the Plutchik’s wheel: serenity, trust, anticipation, apprehension, fear and anger.
iv) Overall experience up to the moment of filling in the questionnaire (scale of five values represented by emoticons  = bad experience;  = great experience)
v) A final open question: ‘What can we do better?’

The analysis of the internal consistency of the questionnaire through an analysis of the closed-ended items showed a high level of reliability (Cronbach’s alpha: patient perspective and preference 13 items > 0.7; patient experience before surgery 20 items > 0.8; patient experience after surgery 11 items > 0.8).

The patient satisfaction questionnaire included demographic data (age, gender, education and region of origin) and assessed patient satisfaction. The 28 items included a first question on overall satisfaction; the items were divided into seven macro-areas: admission and organisation; medical assistance; nursing and other healthcare personnel; services and comfort; religious assistance (if requested); posthospitalisation; and other information. A final question was ‘Would you recommend the hospital to others?’ with a 10-point Likert scale (from ‘Absolutely not” to ‘Absolutely yes’).

### Data collection

An exploratory sample was used, including all orthopaedic patients admitted for surgery during the study period. The patients were recruited at the time of administrative admission for hospitalisation from among those who could understand and consent, speak Italian fluently and write. The data collected were part of the quality of service survey approved by the hospital management and included in the quality surveys in which the patient agreed to participate by signing the consent form at the time of hospital admission. In addition, a trained research assistant asked them for oral consent to participate by explaining the study’s purpose, discussing how participation was voluntary and about the anonymity of data collection.

The fourth author delivered the paper-and-pencil questionnaire to the patient to be filled out on the spot upon arrival in the hospital room (10-min duration) and upon discharge (15-min duration, including the patient satisfaction questionnaire). The same author collected the questionnaire after the patient had filled it in, monitored the completeness of the data and reported all the data on an Excel worksheet for subsequent analysis.

### Data analysis

A score was created for each quantitative item of the questionnaire by coding the item response from ‘1’ if the experience was considered completely negative to ‘5’ if it was considered completely positive. A higher score indicates a positive experience and satisfaction with the hospital patient journey. Quantitative data were analysed with descriptive statistics, including the mean and standard deviation and by analysis of a significant difference between the following a priori established groups: type of surgery (major surgery or minor surgery) and time of the patient journey (at the entrance to the hospital and at discharge). Statistical analyses were performed using SPSS 21.0 (IBM Corp., Armonk, NY, USA). Qualitative data were analysed by the first author by reporting and classifying patient responses to the open question ‘What can we do better?’ Specifically, the content of the responses was classified according to the service quality dimensions of Dagger [27] and Gustavsson [28].

## Results

### Sample characteristics

A total of 255 patients were included in the study; of them, only one patient refused to participate because of the limited time available to prepare for surgery upon entering the hospital. Table 1 shows the main characteristics of the participants. The participants had a mean age of 62 years (SD: 14; range: 18–96), and 80% were over the age of 50. The sample was equally distributed between men and women. The most frequent major surgical operations were knee replacement (53% of major surgery) and hip replacement (29%). The most frequent minor surgical procedures were knee arthroscopy (39% of minor surgery) and shoulder arthroscopy (36%). Of the patients admitted for major surgery, 49% had been admitted to the same hospital in the past, while 70% of the patients who had to undergo minor surgery were being admitted to the hospital for the first time.

**Table 1:** Main characteristics of the patients included in the study

Characteristics	Major Surgery % *(n)*	Minor Surgery % *(n)*	Total % *(n)*
Gender			
Male	55.7 *(88)*	40.6 *(39)*	50.0 *(127)*
Female	44.3 *(70)*	59.3 *(57)*	50.0 *(127)*
Age			
18–30	0.6 *(1)*	10.4 *(10)*	4.3 *(11)*
31–50	10.1 *(16)*	25.0 *(24)*	15.7 *(40)*
51–70	41.1 *(75)*	57.2 *(55)*	42.7 *(120)*
> 70	48.1 *(66)*	7.2 *(7)*	32.6 *(83)*
Highest qualification			
Intermediate	32.2 *(51)*	10.4 *(10)*	24.0 *(61)*
High school	50.0 *(79)*	63.5 *(61)*	55.1 *(140)*
University degree	17.7 *(28)*	26.0 *(25)*	20.8 *(53)*
First admission			
Yes	50.6 *(80)*	70.8 *(68)*	58.2 *(148)*
No	49.3 *(78)*	29.1 *(28)*	41.7 *(106)*
Local health district			
Regional	82.9 *(131)*	81.2 *(78)*	82.2 *(209)*
Extra-regional	17.0 *(27)*	17.7 *(17)*	17.3 *(44)*
Unknown	0.0 *(0)*	1.0 *(1)*	0.3 *(1)*

The evaluation of the patient preferences on the different issues related to the hospital journey that were collected at the beginning of hospitalisation show that the five aspects considered most important for a good hospital journey experience are as follows: ‘Receive the best treatment for the related health conditions’ (Mean: 4.8, SD: 0.4); ‘Have clear instructions on how to prepare for surgery (therapy, fasting, surgery aids)’ (Mean: 4.8, SD: 0.4); ‘Have clear instructions on how to check in at the hospital’ (Mean: 4.7, SD: 0.5); ‘Have clear indications on the treatment pathway I will also have to take’ (Mean: 4.7, SD: 0.5); and ‘Receive explanations from staff in case of waiting’ (Mean: 4.7, SD: 0.5). The least important aspects among those listed are as follows: ‘Have explanations and understand everything that happens to me’ (Mean: 4.0, SD: 0.7); ‘Be involved in all decisions concerning my care’ (Mean: 3.9, SD: 0.8); ‘Feel comfortable in the environments where I have to be’ (Mean: 3.9, SD: 0.9); ‘Wait as little time as possible for a visit or for assistance’ (Mean: 3.9, SD: 0.9); and ‘Have a room where I am not disturbed and with hotel services (TV, landline, etc.)’ (Mean: 3.8, SD: 0.9). When asked if other aspects were important, one participant added ‘Empathic relationship with all the staff’, while another added ‘Admission in a clean facility like this’. No significant differences were found between the major and minor surgery patients.

### Evaluating the patient journey at two different points

All patients completed the quantitative items of the experience questionnaire, which was administered on arrival and on discharge, and the satisfaction questionnaire, which was administered on discharge. On admission, 58% of patients answered the open question ‘What can we do better?’ and 68% answered the same question administered on discharge.

Table 2 reports the answers to the overall questions on patient-reported data, referring to the two moments in which the patients were interviewed.

**Table 2:** Patient-reported data: answers to the overall questions

Patient-reported data	At the time of arrival Mean (SD)			At the time of discharge Mean (SD)		
Question *(scale)*	*Major Surgery* *(n = 158)*	*Minor Surgery* *(n = 96)*	*Total* *(n = 254)*	*Major Surgery* *(n = 158)*	*Minor Surgery* *(n = 96)*	*Total* *(n = 254)*
**Experience**						
How would you assess your overall experience so far? *(1 = negative – 5 = positive)*	4.4 (0.6)	4.3 (0.6)	4.3 (0.6)	4.3 (0.8)	4.5 (0.6)	4.4 (0.7)
**Outcome**						
Are you satisfied with your health? *(1 = not at all – 5 = a great deal)*	3.7 (0.8)	4.0 (0.7)	3.8 (0.8)	4.0 (0.6)	4.2 (0.6)	4.1 (0.6)
Mark with an ‘x’ the level of pain you are experiencing now (0 = absent *–* 10 = the greatest pain)	5.5 (2.7)	2.8 (2.4)	4.5 (2.9)	3.8 (2.6)	2.6 (2.7)	3.4 (2.7)
**Satisfaction**						
Overall, how satisfied are you with your stay at this hospital? *(1 = not at all – 5 = a great deal)*				4.7 (0.6)	4.7 (0.5)	4.7 (0.5)
**Preferences**						
Would you recommend this hospital to others? *(0 = definitely not – 10 = definitely)*				9.4 (1.0)	9.5 (0.8)	9.5 (1.0)

PRO changed between the time of entry and time of discharge, with a different trend between major and minor surgery patients. Upon arrival at the hospital, orthopaedic patients who needed major surgery reported significant pain, here rated on a scale of 0 (absent) to 10 (the strongest pain); this decreased after surgery (Mean: 5.5, SD: 2.7 vs. Mean: 3.8, SD: 2.6). Pain remained constant and not particularly high in minor surgery patients (Mean: 2.8, SD: 2.4 vs. Mean: 2.6, SD: 2.7). The self-reported state of health assessed on a scale of values between 1 (not at all satisfied) to 5 (very satisfied) showed a more evident improvement in patients with major surgery between the time of arrival in the hospital and time of discharge (Mean: 3.7, SD: 0.8 vs., Mean: 4.0, SD: 0.6). Minor surgery patients reported a generally higher level of health than major surgery patients (Mean: 4.0 SD: 0.7 vs. Mean: 4.3, SD: 0.6). In these items, the age group does not seem to be significant.

Regarding the closed-answer items on the overall patient experience, an average of high scores, with a slight difference between the time of entry into the ward and time of discharge, was reported. On discharge, the hospital experience was rated with lower average scores than patient satisfaction. The patient satisfaction relating to hospitalisation showed significant high scores: on a score from 1 to 5, 97% of patients rated 4 (22.8%) or 5 (74.4%). Additionally, 95% of patients would recommend the hospital to other patients.

Table 3 reports how patients’ emotional status changed along the hospital journey. Trust and apprehension were the prevailing emotions at the time of arriving in the ward (respectively 37.8 and 20.5% of patients). Apprehension decreased noticeably among patients after surgery (6.3%), and serenity increased (from 21.7% before surgery to 46.1% at the time of discharge). The change is more evident in major surgery patients: 32.7% of them experienced apprehension or fear before surgery, decreasing to 13.1% at the time of discharge, with an increase in patient serenity from 5.8 to 14.8%.

**Table 3:** Patients’ emotional status

Question	At the time of arrival % *(n)*			At the time of discharge % *(n)*		
What do you feel now?	*Major Surgery*	*Minor Surgery*	*Total*	*Major Surgery*	*Minor Surgery*	*Total*
*Serenity*	22.8 *(36)*	19.8 *(19)*	21.7 *(55)*	45.6 *(72)*	46.9 *(45)*	46.1 *(117)*
*Trust*	32.3 *(51)*	46.9 *(45)*	37.8 *(96)*	25.3 *(40)*	39.6 *(38)*	30.7 *(78)*
*Anticipation*	13.3 *(21)*	21.9 *(21)*	16.5 *(42)*	17.1 *(27)*	8.3 *(8)*	13.8 *(35)*
*Apprehension*	28.5 *(45)*	7.3 *(7)*	20.5 *(52)*	7.0 *(11)*	5.2 *(5)*	6.3 *(16)*
*Fear*	3.2 *(5)*	4.2 *(4)*	3.5 *(9)*	1.3 *(2)*	0.0 *(0)*	*0.8 (2)*
*Anger*	0.0 *(0)*	0.0 *(0)*	0.0 *(0)*	3.8 *(6)*	0.0 *(0)*	2.4 *(6)*

### Detecting areas of improvement by following the patient journey

When analysing the specific items in relation to the time of the journey, the data on experience and satisfaction showed differing information around some key topics. Table 4 shows the experience and satisfaction items that are the most related to the patient’s journey.

**Table 4:** Experience and satisfaction items related to the patient’s journey

Stage of the patient journey	Items	Service quality dimension	Mean	SD
*Outpatient visit and Examination Outpatient Clinic*	*Satisfaction (1 = not at all – 5 = a great deal)*			
	Waiting time between prehospitalisation and hospitalisation	Administrative	4.6	0.7
	Waiting times and handling of incoming documents	Administrative	4.5	0.8
	Courtesy and helpfulness of the administrative acceptance staff	Interpersonal	4.8	0.5
	Clarity of signage inside the university hospital	Environment	4.8	0.5
	*Experience (1 = negative – 5 = positive)*			
	How easy was it to reach the hospital to make the specialist visit with the surgeon?	Administrative	4.7	0.7
	How easy was it to reach the hospital to perform the exams in preparation for surgery (pre-hosp.)?	Administrative	4.6	0.8
	How useful was the information you received to organise hospitalisation?	Administrative	4.5	0.8
	How easy was it to reach the ward?	Administrative	4.6	0.6
*Hospitalisation surgery*	*Satisfaction (1 = not at all – 5 = a great deal)*			
	Quality and cleanliness of the environments	Environment	4.8	0.4
	Availability of your doctor to you and your family	Interpersonal	4.7	0.7
	Professionalism and attention given by your doctor during hospitalisation	Technical	4.8	0.6
	Presence and availability of nurses and healthcare personnel	Interpersonal	4.7	0.7
	Professionalism and competence shown by nurses during care procedures	Technical	4.8	0.6
	Clarity and timeliness in providing information on care and on the state of health	Technical	4.7	0.6
	*Experience from arrival in the ward room to surgery (1 = negative – 5 = positive)*			
	How do you feel inside the room you were assigned to?	Environment	4.3	1.0
	Were you able to be with your family when you wanted?	Family	4.4	0.9
	How useful was the information you received to prepare for surgery?	Technical	4.5	0.8
	Did the doctor explain to you in an understandable way everything you needed to know about surgery, length of stay and the postsurgery period?	Technical	4.3	0.9
	Did the anaesthesiologist explain to you in an understandable way everything you needed to know about surgery and pain treatment?	Technical	4.3	0.9
	Did the doctors give you the time you need?	Interpersonal	4.4	1.0
	Did the nurses give you the time you need?	Interpersonal	4.6	0.7
	Have you been involved in decisions about your care?	Involvement	4.0	1.1
*Postsurgical care and discharge*	*Experience from surgery to discharge (1 = negative – 5 = positive)*			
	Were you able to be with your family when you wanted?	Family	4.7	0.6
	Were the indications on the clinical path after the surgery useful?	Technical	4.4	0.8
	Did the doctors give you the time you need?	Interpersonal	4.3	1.1
	Did the nurses give you the time you need?	Interpersonal	4.5	0.9
	Have you been involved in decisions about your care?	Involvement	4.3	0.9

At the time of discharge, the patient satisfaction items reported high scores for the quality and cleanliness of the environment (Mean: 4.8, SD: 0.4). However, upon entering the ward, the patients rated the comfort of the room with one of the lowest experience scores (Mean: 4.3, SD: 1.0). The answers to the open questions show the reason for this: the patients wished to have a TV inside the wards and to have larger wards to move more easily with the orthopaedic aids they had to manage (wheelchair, crutches, etc.). One patient suggested the following solution: ‘Small hospital room for physiotherapy: creation of a dedicated space’ (Code: ORTO 63).

In the ‘Satisfaction’ items, patients recognised a high level of professionalism and competence in the healthcare staff (Mean: 4.8, SD: 0.6). However, in the ‘Experience’ questionnaire, the items concerning information received before surgery showed some of the lowest scores. The score on the information received to organise the hospitalisation and prepare for surgery was rated at 4.5 (SD: 0.8). Understandable explanations given before surgery by the doctor of everything the patient needed to know about surgery, length of stay and the postsurgery period was rated 4.3 (SD: 0.9). The same result was recorded for understandable explanations given by the anaesthesiologist on everything the patient needed to know about surgery and pain treatment. The answers to open questions showed that 29 patients would have liked more information concerning the different aspects of hospitalisation, including the necessary aids for surgery and postsurgery path. Two patients emphasised the need for more communication with family members when the patient was in the operating theatre. One patient expressed how this issue can always be improved: ‘In my opinion, improve the information given to patients on the path they have to take inside the hospital. I have been hospitalised five times and I always see an improvement, thanks for everything’ (Code: DS36).

The patient satisfaction questionnaire reported a high score on the availability of doctors and nursing and care staff (Mean: 4.7, SD: 0.7). For the experience items, the patients rated these aspects at 4.4 and 4.6, respectively, at the time of entering the ward. The average score decreased to 4.3 and 4.5, respectively, regarding the postsurgery stay. More specific data emerged from the open questions. The patients reported the need for more of a presence of and contact with doctors (38 quotes) and nurses (21 quotes), and this need was reported in particular regarding the postsurgery stay: ‘More time spent by staff in the postoperative period’ (Code: ORTO2). Twenty-one patients reported a lack of interaction with healthcare staff as a staff shortage problem: ‘Nurses are very professional and well trained, but there should be more of them’ (Code: DS37); ‘too few nurses during the shift to answer the call bells quickly’ (Code: ORTO151). Other patients added that the presence of so many students decreased their confidence in being properly cared for. For example, one patient said, ‘Stay longer with the patient without rushing, too many students unable to solve certain problems and too few nurses and doctors’ (Code: ORTO 116).

The question ‘Were you involved in decisions about your care?’ obtained the lowest score. Specifically, the average rating was 4.0 upon arrival in the ward and increased to 4.3 upon discharge. However, only one participant suggested greater patient involvement.

The satisfaction score on waiting times and admissions procedures was among the lowest (Mean: 4.5, SD: 0.8). The reasons for these scores were expanded by the answers to the open questions captured immediately after entering the ward: 53 patients reported that the waiting times between arrival at the hospital, admission procedures and room assignment were too long. One patient pointed out that hospital discharges and new entries needed to be better coordinated; another suggested that the patient should not come too early in the morning if admission was scheduled during the day; some patients asked for a reduction in the time between entering the hospital and actually entering the operating theatre.

Some hidden but not openly stated needs for adaptation by the patient to hospital rules are evident in this quote: ‘I found everything well, no complaints, I understood that having a relative’s personal assistance is impossible but I would have liked it’ (Code: ORTO26). In the presurgery period, the patients reported the desire for family members to be nearby when they wanted (Mean: 4.4, SD: 0.9). The score increased in the postoperative period (Mean: 4.7, SD: 0.6), and only nine patients stated they wanted more time with their families, with more flexible visiting hours and with their presence before surgery.

Although unsolicited, feedback on what works, in addition to what needs to be improved, was given. For example, one patient reported, ‘I did not expect to find such a comfortable environment with such professionalism from all the staff. Nothing is perfect, therefore everything is perfectible, but here, in this hospital, we are at a good point’ (Code: ORTO16). Another said, ‘Nothing to improve, on the contrary I would like to point out the particular care, attention and professionalism of the student F.A.’ (Code: ORTO33).

Table 5 reports the improvements suggested by the patients collected at the two different points of their journey.

**Table 5:** Patient-reported improvements

What can we do better?	Service quality dimensions	At time of arrival % *(n)*	At time of discharge % *(n)*	Total % *(n)*
Room comfort (TV, spaciousness, temperature, etc.)	Environment	28.3 *(72)*	19.3 *(49)*	47.6 *(121)*
Management of prehospitalisation	Administrative	5.1 *(13)*	–	5.1 *(13)*
Waiting times from the moment of arrival at the hospital to the moment of entering the operating theatre	Administrative	14.6 *(37)*	6.3 *(16)*	20.9 *(53)*
Availability of nursing staff	Interpersonal	3.5 *(9)*	11.4 *(29)*	15.0 *(38)*
Availability of medical staff	Interpersonal	2.0 *(5)*	8.3 *(21)*	10.2 *(26)*
Information on the clinical path	Technical	4.7 *(12)*	6.7 *(17)*	11.4 *(29)*
More frequent physiotherapy	Technical	–	11.0 *(28)*	11.0 *(28)*
Better pain control	Technical	–	7.9 *(20)*	7.9 *(20)*

## Discussion

Numerous studies have explored how different types of data collected directly from patients can improve the quality of care, while few studies have analysed whether the data reported by patients on a cross-hospital process can be useful to improve the process itself [29, 30]. The current study was designed to explore whether patient-reported data, specifically experience data, can identify areas of improvement within the hospital and between the different units crossed by the patient journey.

By timely and simultaneously gathering preferences, satisfaction, outcomes and experience data, it is possible to have a complete picture of the hospital patient journey. In particular, the satisfaction and preference data measure the levels of importance given to different aspects by the patient—*what* is important for the patient experience—and outcomes data show the patient’s circumstances and present conditions—*why* it is important for the patient. Satisfaction data may not reveal pathology-related needs, while patient experience data can detect important areas of improvement along the hospital journey. In the current study, for instance, the score of preference for room comfort was one of the lowest items (Mean: 3.8). However, by answering to the open question on what can be improved, when the patient entered the room, he judged it not very comfortable (Mean: 4.3) because of the lack of space for movement with orthopaedic aids. The satisfaction item cannot capture this information (average score of 4.8 on the quality and cleanliness of the environments).

These same results also show how at the beginning of the journey, patients might consider an issue as not critical, but while living the hospital experience, it becomes important. As another example, although on admission, patients declared that waiting as little time as possible for a visit or for assistance was one of the least important aspects (Mean: 3.9), the satisfaction score on waiting times and admissions procedures was among the lowest (Mean: 4.5). Timely experience data show how as soon as the patient enters the room, he or she clearly remembers having waited too long from the moment of admission and suggests a better organisation of the hospitalisation.

Numerous factors can influence the patient experience. In the presurgery stage, trust and apprehension are the prevailing emotions (37.8 and 20.5% of patients, respectively), and the patient seems to lack knowledge of what to do. The experience data show that the scores related to information received to organise the hospitalisation and prepare for surgery and explanations given before surgery are very low. Improving information concerning the different aspects of hospitalisation, including the necessary aids for surgery and the postsurgery path, emerged as a fundamental need.

Even if related to a very specific case, the results of the current study show that patients do not have the technical competence to predict their needs before and after surgery; thus, nursing competence is needed to effectively anticipate patient needs and attend to the organisation of patient journeys to improve experiences of care. These data support the claim of a recent NHS report in which nurses are shown to play an essential role in the way in which data are collected, interpreted and used to improve care [10].

Because clinical conditions and the context of care change rapidly within a single hospital stay for surgery, capturing data at key moments in the journey, rather than at the end, can better represent the patient’s experience [31]. In particular, analysing the answers to the open question ‘What can we do better?’ allows for understanding what happened to the patient that may have influenced his or her experience (e.g., apprehension and pain before surgery, pathology and age-related needs, fast-track recovery, waiting without entertainment, etc.). Moreover, one patient may reveal the important needs made impossible by circumstances (e.g., need of having a family member being close to the patient before surgery made impossible by hospital organisation). For example, when redesigning fast-track recovery from major orthopaedic surgery, significant touchpoints for the patient should be treated with respect to his or her need for interaction with professionals, his or her emotional state and social conditions and by considering the changing circumstances he or she will face along the journey [19, 32, 33]. In particular, the emotional state should be better explored to understand how this variable affects patient experience along the journey and to improve ways of interacting with the patient: by giving more information, by offering support or simply by accompanying him or her in critical moments of the presurgery period.

In the present study, to encourage patient participation, the authors decided to ask a few questions at two critical moments of the journey: at the arrival before surgery and at discharge. Moreover, despite the older population involved, the simplicity of the questionnaire, which even used emoticons, made it possible to capture the experience of those patients who were able to read and write. In this way, a high rate of responses was achieved. Nevertheless, further studies should investigate how to collect real-time feedback from those who are unable to describe their own experience [34]. Moreover, because data were collected in paper format, the process of returning data to the management team and front-line professionals to stimulate quality improvement slowed down because of the necessary data analysis times. The challenges in handling real-time big data collection and storage in health information systems will bring new advancements in the continuous improvement process by immediately returning the patient-reported data at all organisational levels. These data should help redesign hospital care processes at the top and middle management levels in an integrated and patient-centred way. At the front-line level, healthcare professionals can immediately make corrections with micro-interventions, fixing the way of giving attention to the patient by focusing on his or her experiences.

In present study, patient-reported data on satisfaction and experience were significantly positive in almost all the items investigated, with an average score between 4 and 5 on a scale of 1 to 5. This result is in line with the literature showing that little variation occurs in the answers to questions about the quality of care with high patient satisfaction scores [35, 36]. Further studies should investigate if the asymmetrical relationship between the health care professional and patient [37], the vulnerable situation of a patient [34] and the primary need to solve a clinical problem and fear of surgery that are deemed more important than anything else could result in high and undistributed response rates.

When asking the patient ‘What can we do better?’ the question assumes that the patient only identifies what does not work. However, patients are also able to report what worked well. Managers and health care teams should study which factors, from the patient’s point of view, determine a good experience and must be supported. Further studies should analyse whether positive patient-reported data may explain what factors produce a good patient journey experience and how they may reinforce the quality improvement solutions adopted and, hence, influence health professionals’ behaviour [38].

The results of the current study emphasise that personalised medicine should no longer only refer to the targeted therapy. This requires management teams to be able to customise the patient journey and identify different patient profiles, which should not be reduced to the clinical pathway.

The limitations of the current study are manifold; in large part, they are connected to the nature of the original project, which aimed to produce local actionable improvements in the setting. First, the results cannot be generalised: the study was conducted in a single hospital and only on how the orthopaedic surgical path; this influences the significance and transferability of the results. However, the study aimed to provide useful insights to hospital management to promote a review of the processes in a real patient-centred way. In particular, the results offer a stimulus for the debate on the use of patient experience data for the design of service delivery. Second, the orthopaedic surgical path is very different, for example, from the oncological one. Specifically, the orthopaedic surgical journey is generally shorter, has a beginning and an end and does not extend over time with a worsening of the initial clinical conditions. This aspect could influence the results and methodology should be tested on different clinical pathways, in particular by considering chronic pathologies. Finally, the authors preferred to use a questionnaire with relatively few questions instead of using the validated ones already present in the literature. The choice was made to favour real-time data collection and the patient’s response rate to the questions. These objectives have been achieved, and future studies will have to validate the single items for patients with different pathologies. However, by considering the very different and complex contexts in which each hospital operates, the literature will increasingly have to consider single longitudinal studies by starting from the analysis of the patient journey that takes place in each hospital.

Several issues would benefit from further exploration, including the impact of the patient-healthcare staff relationship in the hospital journey experience; the opportunity of bringing patients’ and professionals’ experiences together for joint knowledge of improvement solutions; and the study of new methodology to capture the real-time experiences of vulnerable patients.

## Conclusion

Providing customers with quality experiences is a key competitive advantage in a range of service sectors, including the healthcare service. Measuring patient experiences is a practice increasingly used, and researchers and managers are seeking to understand how to use these measures to improve service delivery [39–41].

The current study provides insights for healthcare practitioners caring for patients in hospitals and those responsible for planning and designing the hospital patient journey. By contributing to the literature on how patient-reported data could be collected and used in hospital quality improvement, it also opens the debate about the use of real-time focused data when capturing experiences from vulnerable patients. Furthermore, the present study asks for a more positive perspective on patients’ data that can be used not only to detect what does not work, but also what is working well.

In different clinical settings, further studies should explore how to effectively use patient-reported data to improve hospital processes, profile patients’ needs and identify appropriate methodologies to capture the experiences of vulnerable patients. These topics may offer new frontiers of research to achieve a patient-centred healthcare system.

## Supplementary Information

**Additional file 1.** Questionnaire. Questionnaire used for the study.

## Data Availability

The datasets used and/or analysed during the current study are available from the corresponding author upon reasonable request.

## Abbreviations

PRO: Patient-reported outcomes
PRE: Patient-reported experience
PRS: Patient-reported satisfaction
PRP: Patient-reported preferences
N: Number count
SD: Standard deviation
NHS: National Health Service (UK)
