# Supplementary material for: What does the patient have to say? Valuing the patient experience to improve the patient journey
Source: BMC Health Serv Res. 2021 Apr 15;21:347. doi: 10.1186/s12913-021-06341-3 (PMC8048032; doi:10.1186/s12913-021-06341-3)
Supplement: Supplementary file 1 — Additional file 1. Questionnaire. Questionnaire used for the study. [file 12913_2021_6341_MOESM1_ESM.docx]

**Questionnaire**

**Your experience in our hospital: what is important to you?**

We are interested in your experience to improve our service

How old are you?

Mark with 'x' the level of pain you now feel : 0 absent – 10 very high

Are you satisfied with your state of health? (1 not at all – 5 completely)

How much each of these aspects is important to you (1 few – 5 a lot)

- To have clear instructions on how to present myself at the hospital
- To receive explanations from staff when waiting
- To have clear indications about the course of treatment I will have to follow
- Have clear indications on how to prepare myself for the surgery (therapy, fasting, surgery material)
- Receive the best treatment for my condition
- To feel no pain
- To be confident that I will be able to resume my daily activities independently once I leave hospital.
- Trusting the people who take care of me
- Have explanations and understand everything that happens to me
- To be involved in all decisions concerning my care
- To feel comfortable in the environments I need to be in.
- Have a room where you will not be disturbed and with television, WIFI, telephone, etc..
- Waiting as little time as possible for a visit or care intervention
- Other: Insert here other aspects that you consider important

**Your experience in our hospital: before admission**

We are interested in your experience from the first outpatient visit to the day of surgery.

- How did you book your first appointment with the orthopaedic specialist? In person at the hospital, By telephone booking, By online booking
- Where did you have your first visit with the orthopaedic specialist? (Specify city and name of facility)
- Where did you perform examinations in preparation for surgery? (Specify city and name of facility)
- How easy was it to get to the facility to have the specialist consultation with the surgeon?
- How did you feel in the environment where you had your specialist consultation with the surgeon?
- How easy was it to get to the facility for pre-hospital examinations?
- How did you feel in the environment where you had your pre-surgery examinations (pre-hospitalisation)?
- How helpful was the information you received in preparing for surgery?
- How helpful was the information you received in organising your hospitalisation?
- How easy was it to get to the ward?
- How do you find yourself inside the room to which you have been assigned?

**Your experience in our hospital: admission**

We are interested in your experience from the moment you arrive at the hospital to the time of surgery.

Answer to each of these questions 1 not at all – 5 completely

- Did the anaesthetist explain to you in an understandable way everything you needed to know about surgery and pain management?
- Did the doctor explain to you in an understandable way everything you needed to know about the surgery, the length of your stay and the post-operative period?
- Were you involved in decisions about your care?
- Did you feel that your problems were taken seriously?
- Were you able to be with your family when you wanted to be?
- Were you treated with respect, courtesy and care by the doctors?
- Did you trust the doctors who treated you?
- Did the doctors give you the necessary time?
- Did the nurses explain the tasks to be carried out to you in an understandable way?
- Were you treated with respect, courtesy and care by the nurses?
- Did you trust the nurses who took care of you?
- Did the nurses give you the necessary time?

What do you feel now? Serenity, Confidence, Expectation, Concern, Fear, Anger

Write here what you feel in your own words:

How do you generally assess your experience so far?

What can we do better? Write here what you would like to see improved.

**Your experience in our hospital: after surgery**

We are interested in your experience during your stay after surgery

Mark with 'x' the level of pain you now feel : 0 absent – 10 very high

- Was the guidance you received on the clinical pathway after surgery helpful?
- Were you involved in decisions about your care?
- Did you feel that your problems were taken seriously?
- Were you able to be with your family when you wanted to be?
- Were you treated with respect, courtesy and care by the doctors?
- Did you trust the doctors who treated you?
- Did the doctors give you the necessary time?
- Did the nurses explain the tasks to be carried out in an understandable way?
- Were you treated with respect, courtesy and attention by the nurses?
- Did you trust the nurses who took care of you?
- Did the nurses give you the necessary time?

What do you feel now? Serenity, Confidence, Expectation, Concern, Fear, Anger

Write here what you feel in your own words:

What is your overall assessment of your experience at this hospital?

Are you satisfied with your state of health?

What can we do better? Write here what you would like to see improved.
